# Pachychoroid disease and its association with retinal vein occlusion: a case–control study

**DOI:** 10.1038/s41598-021-99115-0

**Published:** 2021-10-06

**Authors:** Leonie F. Keidel, Sarah Zwingelberg, Benedikt Schworm, Nikolaus Luft, Tina Herold, Siegfried G. Priglinger, Jakob Siedlecki

**Affiliations:** 1 Department of Ophthalmology, Ludwig-Maximilians-University, Munich, Germany; 2 Department of Ophthalmology, University Hospital Cologne, Cologne, Germany

**Keywords:** Retinal diseases, Uveal diseases, Scleral diseases

## Abstract

The development of a retinal vein occlusion (RVO) is multifactorial. This study investigates pachychoroid as a risk factor for RVO or as an entity sharing common pathophysiology with RVO. A database screening at the University Eye Hospital, Ludwig-Maximilian University Munich, Germany was performed for patients diagnosed with central or branch RVO (CRVO/BRVO). In every patient a complete ophthalmologic examination was performed, including posterior segment enhanced depth spectral domain optical coherence tomography (EDI-SD-OCT). The SD-OCT scans of respective partner eyes without history of RVO were compared to an age- and refraction-matched, randomly recruited normal control group. In total, 312 eyes of 312 patients were included in this study, with 162 eyes in the RVO and 150 eyes in the control group. A significantly higher subfoveal choroidal thickness (SFCT) was found in the RVO (310.3 ± 72.5 (94 to 583) µm) as compared to the control group (237.0 ± 99.0 (62 to 498); p < 0.00001). Moreover, the RVO group showed a significantly higher prevalence of a symptomatic pachychoroid (22 vs. 9 eyes; odds ratio: 2.46; 95 CI: 1.10 to 5.53; p = 0.029). Since pachychoroid disease represents a bilateral entity, it might be a risk factor for RVO, or share risk factors with RVO.

## Introduction

In central retinal vein occlusions (CRVO), multiple risk factors have been described, including arterial hypertension, diabetes mellitus, increased intraocular pressure and hypercoaguability^1^. Since, however, in many patients none of the above-mentioned factors can be established, most authors deem the pathophysiology to be multifactorial^1^ and not yet known risk factors have to be taken into consideration.

It is commonly assumed that the occlusion of the central retinal vein follows a thrombotic event, mostly at the passage through the lamina cribrosa. In this segment, the central retinal vein narrows, blood flow is more turbulent and thrombus formation is therefore promoted. Thus, any factor contributing to narrowing of the vein and/or to an increase in turbulence of flow may increase the likelihood of a retinal vein occlusion (RVO)^2^. Recently, Nagia et al. found peripapillary pachychoroid features in partner eyes of eyes with non-arteriitic anterior ischemic optic neuropathy (NAION), leading to the hypothesis that a thicker choroid may be associated with a higher risk for the development of NAION due to narrowing of the optic sheath^3^.

Therefore, this study was designed to investigate choroidal morphology, thickness and associated pachychoroid phenotypes in patients with CRVO or branch retinal vein occlusion (BRVO).

## Material and methods

For this retrospective case–control study, all consecutive eyes presenting with CRVO or BRVO at the University Eye Hospital of the Ludwig Maximilian-University Munich, Germany, between January 2017 and January 2019 were included into this study from the clinic’s electronic database. These were then matched by age and spherical refraction to a random control group from the same timespan. Institutional review board approval of the Ludwig-Maximilian University, Munich was obtained for this retrospective chart review, and the study adhered to the tenets of the Declaration of Helsinki. All patients provided written informed consent prior to any study-related procedure.

Epidemiological data was obtained from each patient, including age, gender, previous ocular comorbidities or procedures and objective refraction-based Snellen chart visual acuity which was later converted to logMAR for analysis. Patients’ spherical equivalents and spheres were obtained by the auto refractometer Nidek AR-1 s (Oculus GmbH, Wetzlar, Germany).

### Imaging

Imaging (all on Spectralis HRA + OCT, Heidelberg Engineering, Heidelberg, Germany) was performed after pupil dilation with topical tropicamide 1% and phenylephrine 2.5%. It included an acquisition of a enhanced depth imaging macular volume scan consisting of 49 equally spaced B-scans covering 20 × 20 degrees centered on the fovea. Fluorescein (FA) and/or indocyanine green (ICG) angiography scans as well as OCT angiography were performed at the investigator’s discretion.

Subfoveal and sublesional choroidal thickness (SFCT, SLCT) were assessed by manual measurements of the distance between the Bruch’s membrane and the choroidal–scleral interface below the foveola or below the center of a pachychoroid lesion, respectively. Pachychoroid was defined as: (i) SFCT > 350 µm with a characteristic pachyvessel configuration in Haller’s layer and an attenuation of the choriocapillaris^4^. Symptomatic pachychoroid was defined as the presence of either pachychoroidal pigment epitheliopathy (PPE), central serous chorioretinopathy (CSCR), pachychoroid neovasculopathy (PNV) or pachychoroid aneurysmal type 1 choroidal neovascularization (formerly polypoidal choroidal vasculopathy) (PAT1)^4^. Changes were considered PPE when RPE changes above a focally thickened choroid were observed, including RPE mottling as well as irregular areas of RPE arrosion or elevation without the appearance of soft drusen or reticular pseudodrusen as seen in age related macular degeneration^4^. CSCR was defined as a serous neurosensory retinal detachment with or without serous pigment epithelial detachment (PED)^4^. In PNV, a CNV formation atop of a thickened choroid had to be visualized on OCT and confirmed by fluorescein and indocyanine green angiography with evidence of a staining plaque on ICGA as well as flow within the CNV on OCT angiography^4^.

Changes were considered a PAT1 when RPE detachments associated with choroidal polypoidal/aneurysmal signs were present on OCT and ICGA showing the typical branching vascular network (BVN) appearance with terminal aneurysmal dilatations^5^.

### Statistical analysis

All data were gathered and analyzed in Microsoft Excel spreadsheets (Version 16.23 for Mac; Microsoft, Redmond, WA, USA). Statistical analysis was performed in SPSS Statistics 26 (IBM Germany GmbH, Ehningen, Germany). The level to indicate statistical significance was defined as p < 0.05. The Kolmogorov–Smirnov tests were employed to test for normal distribution. Statistical analyses of inter-group differences were performed using the independent two-tailed Student t-test and the Man Whitney U Test, as well as Fisher's exact test.

## Results

### Patient characteristics

The RVO group included 162 eyes of 162 patients with a male to female ratio of 94:68 and a mean age of 66.2 ± 13.8 (22 to 87) years. There were 107 patients with CRVO (66.1%) and 55 patients with BRVO (33.9%). The control group consisted of 150 eyes of 150 patients with a male to female ratio of 68:82 and a mean age of 66.3 ± 16.5 (23 to 95) years. Detailed patient characteristics can be found in Table 1.

**Table 1:** Demographic characteristics and the distribution of pachychoroidal features in the RVO group compared to the control group.

	RVO partner eyes (mean ± SD; range)	Control group (mean ± SD; range)	p
No. of eyes (n)	162	150	
No. of patients (n)	162	150	
Gender	94 m/68 f	68 m/82 f	
Mean age (yeras)	66.2 ± 13.8 (22 to 87)	66.3 ± 16.5 (23 to 95)	p = 0.96
SE (D)	0.21 ± 2.1	0.10 ± 3.3	p = 0.20
Subfoveal choroidal thickness (µm)	310.3 ± 72.5 (94 to 583)	237.0 ± 99.0 (62 to 498)	p < 0.00001
Symptomatic pachychoroid	22 (13.6%)	9 (6.0%)	p = 0.036
**Pachychoroid stage**			
Stage 1 (PPE)	19 (86.4%)	7 (77.8%)	p = 0.025
Stage 2 (CRCS)	2 (9.1%)	2 (22.2%)	p = 1.0
Stage 3 (PNV)	1 (4.5%)	0	p=1.0
Stage 4 (PAT1)	0	0	

There was no difference in mean age (p = 0.96) and spherical equivalent between the two groups (RVO group 0.21 ± 2.1 vs. control group 0.10 ± 3.3 D, p = 0.20). Even when separating CRVO and BRVO patients, there was still no difference in mean age (p = 0.40) in the CRVO group, as well as in the BRVO group (p = 0.55) compared to the control group. Also there was no difference in spherical equivalent in the CRVO group and BRVO group compared to the normal control group (p = 0.60 and p = 0.58) and there was no difference in sphere in the CRVO group and BRVO group compared to the normal control group (p = 0.76 and p = 0.75).

### SD-OCT image analysis

**Figure 1:**
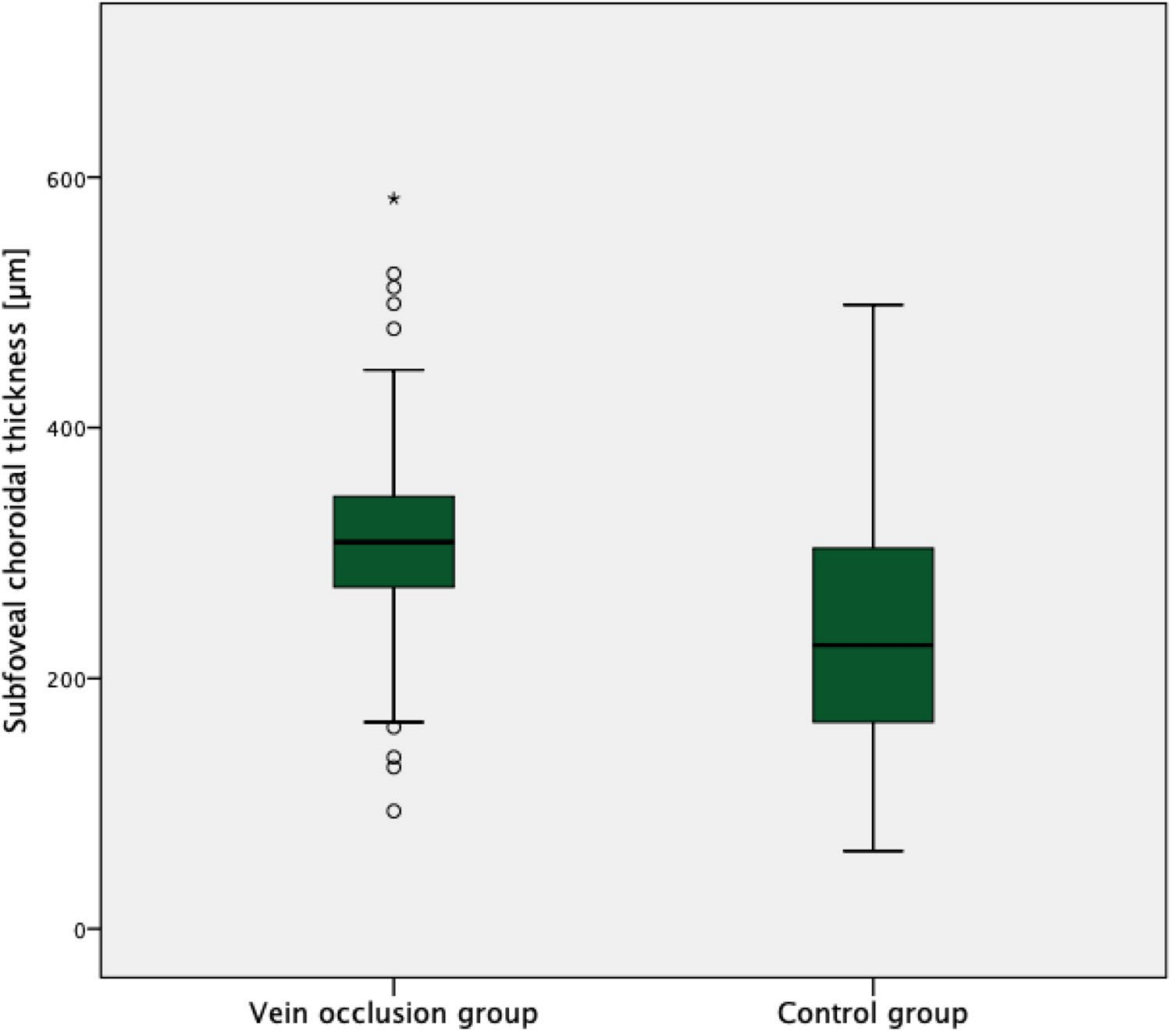
SFCT in the RVO and control group. Boxplots showing significantly increased SFCT in the RVO group compared with the control group.

**Figure 2:**
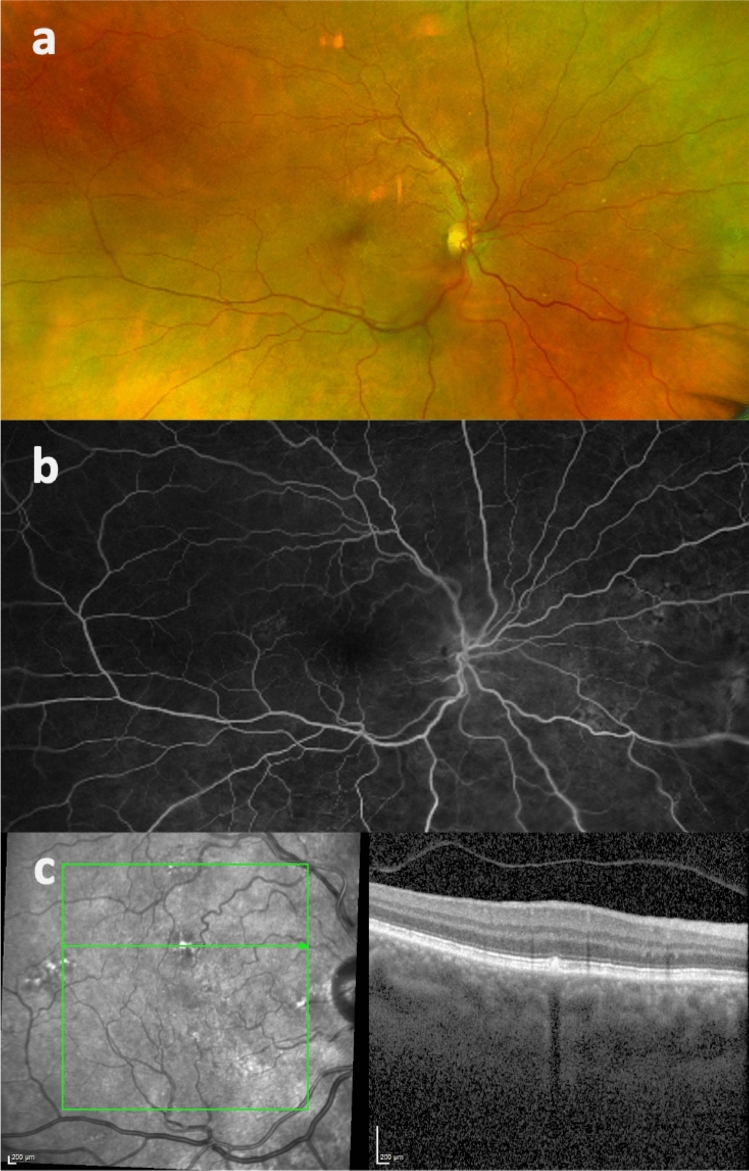
Multimodal imaging of a partner eye of an eye with acute CRVO. Fundus photography (**a**), fluoresceine angiography (**b**) (Optos system, Dunfemline, UK) and OCT-B scan (**c**) at the level of the PPE.

**Figure 3:**
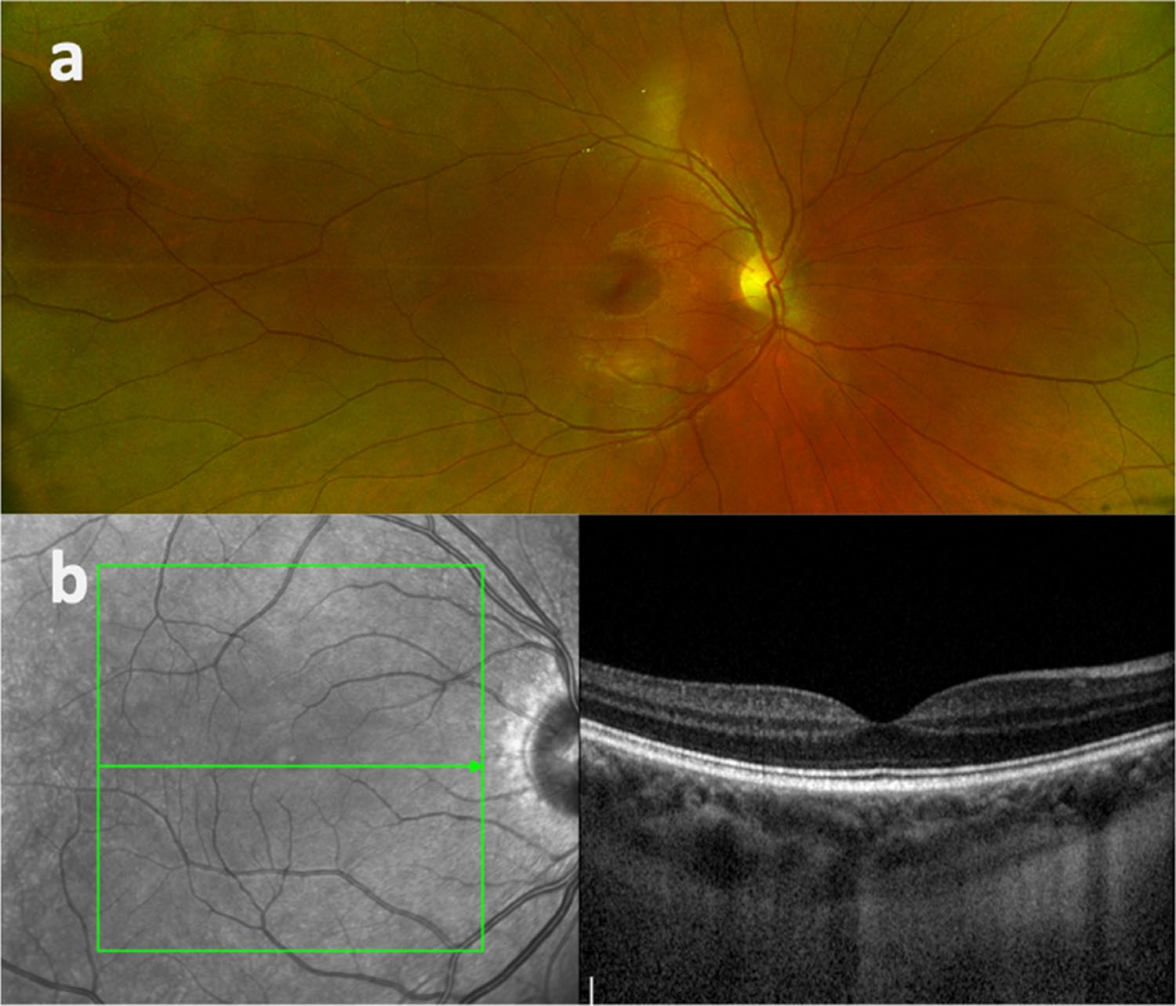
Multimodal imaging of a normal control patient. Fundus photography (**a**) (Optos system, Dunfermline, UK) and OCT B-scan (**b**).

A significantly higher SFCT was found in the RVO (310.3 ± 72.5 (94 to 583) µm) as compared to the control group (237.0 ± 99.0 (62 to 498); p < 0.00001) (Fig. 1). Moreover, the RVO group showed a significantly higher prevalence of a symptomatic pachychoroid (22 vs. 9 eyes; p = 0.036), which was PPE in 86.4% (19 eyes) (Fig. 2), CSC in 9.1% (two eyes) and PNV in 4.5% (one eye) of cases. In the control group 77.8% (7 eyes) showed PPE and 22.2% (two eyes) CSC (Fig. 3). The Odds ratio for symptomatic pachychoroid was 2.46 (95 CI: 1.10 to 5.53; p = 0.029).

**Figure 4:**
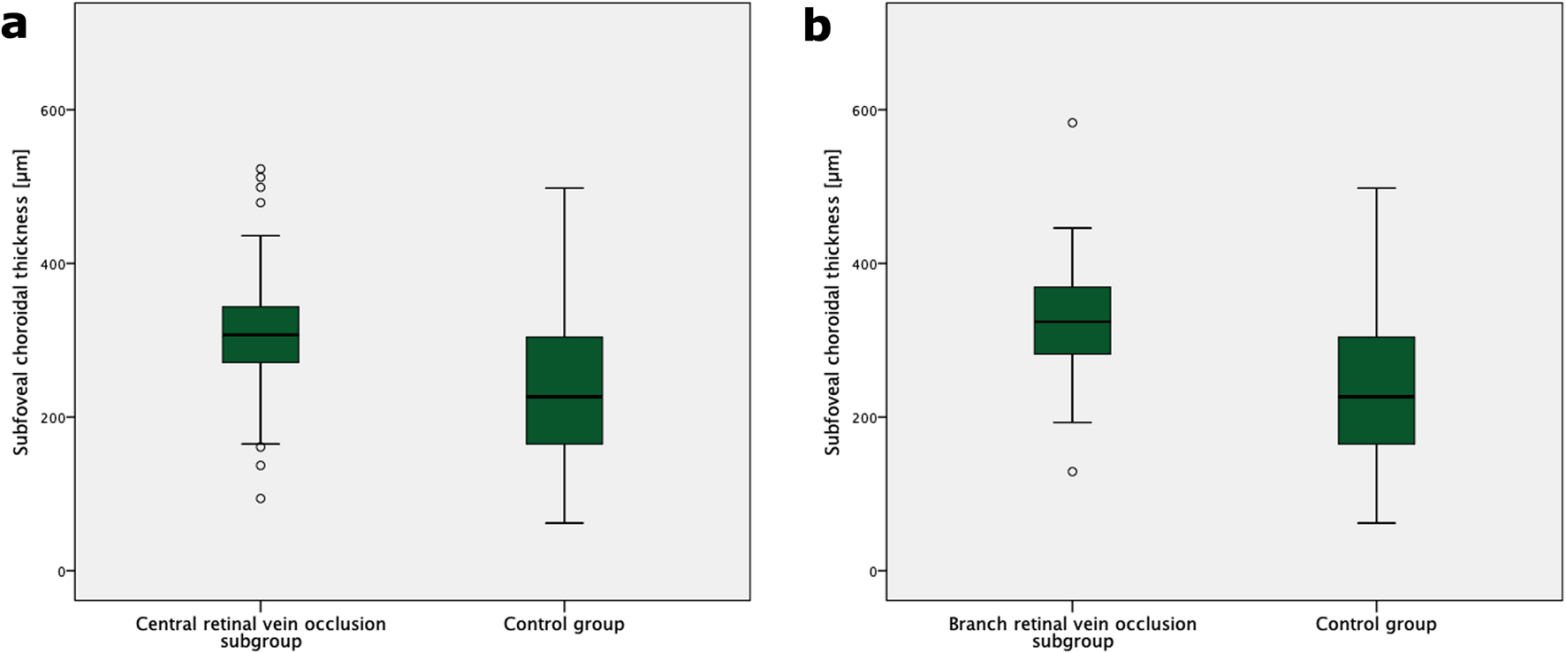
SFCT in the CRVO and BRVO subgroups and the control group. Boxplots showing significantly increased SFCT in the CRVO (**a**) and BRVO (**b**) subgroups compared with the control group.

When evaluating CRVO and RVO separately, there were 15 cases of symptomatic pachychoroid in the CRVO group (68.2%) and 7 cases of symptomatic pachychoroid in the BRVO group (31.8%). SFCT was still significantly higher in the CRVO (304.9 µm ± 71.1 µm, p < 0.00001, Fig. 4a) and BRVO (320.9 µm ± 74.8 µm, p < 0.00001, Fig. 4b) subgroup compared to the control group.

**Figure 5:**
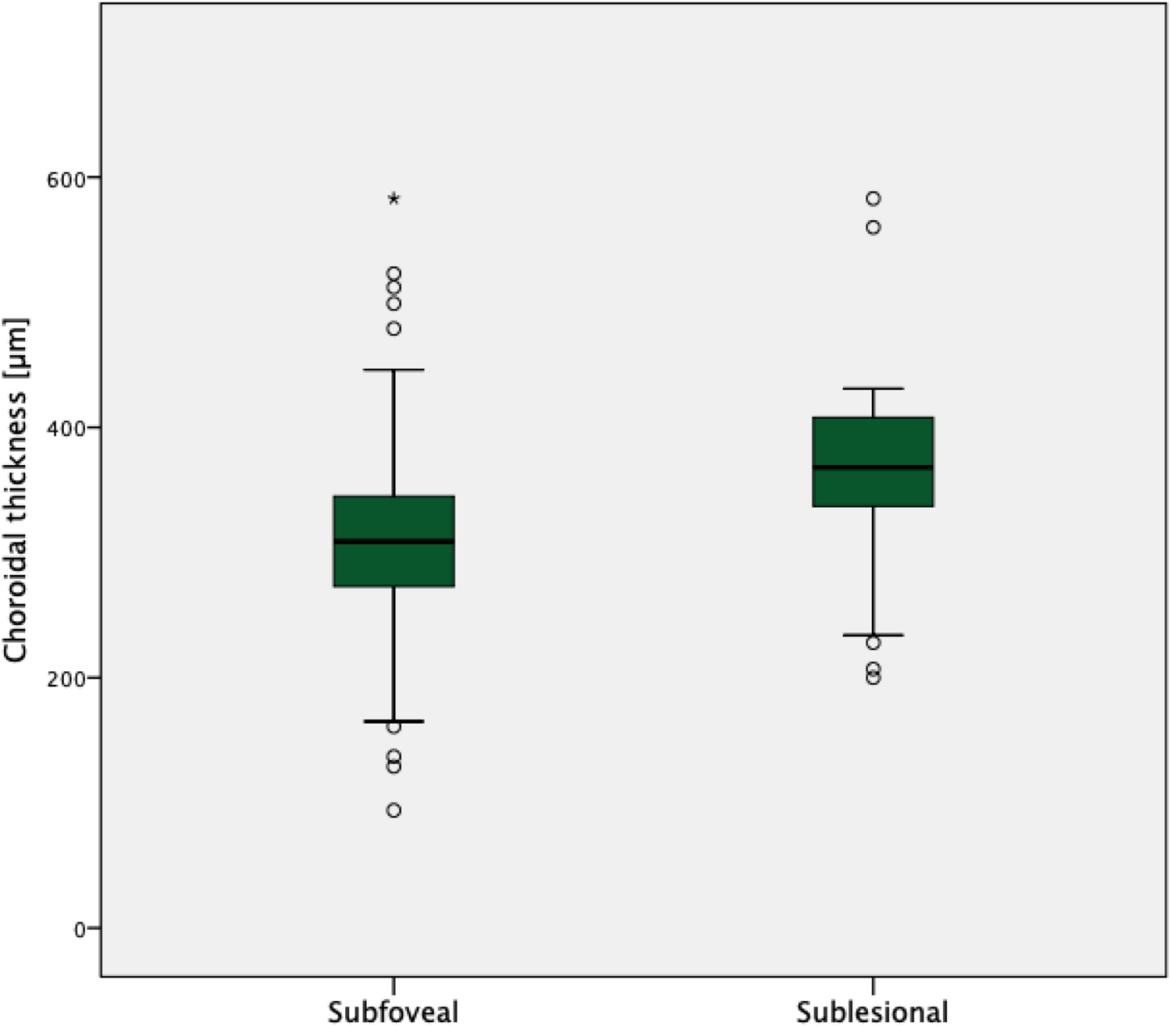
SLCT and SFCT in the RVO group. Boxplot showing increased SLCT in the RVO group compared with SFCT as evidence of the pachychoroidal entity.

In both groups, the SLCT exceeded the SFCT as a proof for its pachychoroid etiology (SLCT RVO group: 365.6 µm ± 95.5 µm; p = 0.015; SLCT control group: 357 µm ± 68.2 µm; p = 0.014, Fig. 5).

Table 1 shows the distribution of pachychoroid features in the RVO group compared to the control group.

## Discussion

The present study found a significantly higher SFCT in patients (not eyes) suffering from RVO as compared to an age- and refraction-matched control group. Moreover, patients with RVO showed a significantly higher prevalence of a symptomatic pachychoroid, indicating that pachychoroid phenotypes might be associated with RVO or that both entities might share similar pathophysiologic mechanisms. Since pachychoroid disorders and their anatomy (e.g. SFCT) are thought to be bilateral, this analysis explicitly included the partner eyes of eyes with RVO to avoid confounders induced by the RVO itself.

The newly characterized spectrum of “pachychoroid disorders” contains PPEs, CSCR, PNV, PAT1 and pachychoroid geographic atrophy^6^. It has been suggested by our group that these features represent a continuum of one disease in four stages in exactly this order, rather than separate entities^7^. They all describe a phenotype characterized by an attenuation of the choriocapillaris and Sattler’s layer overlying focally or generally dilated choroidal veins in Haller’s layer (pachyvessels). The mechanisms that underlie the formation of serous PEDs in the first place are as of yet unclear. The attenuation of the choriocapillaris may create an ischemic environment and the choroidal thickening in Haller’s layer, caused by an enhanced perfusion in this area may increase hydrostatic pressure. Both factors challenge the RPE-Bruch complex and may damage the overlying structures mechanically, inducing atrophic changes in the RPE cells and focal breaks in Bruch’s membrane (PPE stadium). Overcoming the tight junctions between RPE cells may result in focal or diffuse leakage in the subretinal space (CSCR stadium)^4,8^.

Since pachychoroid spectrum diseases represent a new entity, many associations of these disorders with well-established vascular diseases remain to be investigated.

Two explanations for the association of pachychoroid spectrum disorders with RVO can be given.

Recently, involvement of the optic nerve head in choroidal thickening has been described and termed peripapillary pachychoroid^9^. Nagia et al.^3^ published work connecting pachychoroid with non arteriitic ischemic optic neuropathy (NAION). In their study, peripapillary pachychoroid features were found in NAION eyes and their respective partner eyes, leading to the hypothesis that a thicker choroid may be associated with a higher risk for the development of NAION^3^. Alterations of the choroidal volume may create a “compartment syndrome” of the optic nerve with a subsequent thinning of its blood supply through the compression of the afferent artery^3^. In our group pachychoroid features were found in partner eyes of eyes with vein occlusions. The concept of Nagia et al. may as well be transferred to the origin of RVOs. Any factor contributing to narrowing of the vein and/or increasing turbulences of flow may increase the likelihood of a RVO^2^. The thickened choroid found in RVO eyes may further compress the retinal vein at its passage through the lamina cribrosa and consequently induce venous stasis and turbulences of blood flow in this segment. Venous stasis as one factor of the Virchow’s triad is held responsible for the formation of blood clots^1^. The increase in blood stasis in eyes with pachychoroidal features may therefore predispose to the formation of thrombi in a retinal vein and ultimately lead to a RVO.

Second, recent data suggest that scleral anatomy might be both altered in pachychoroid disease and RVO, and thus both diseases could share the same etiology, i.e. an altered sclera, either thickened, more rigid, or both.

The reason for the development of a pachychoroid still remains unclear and only hypotheses have been made so far. Recent studies revealed that vortex veins in CSCR patients appear asymmetrically dilated and collateral formations due to venous anastomosis have been identified on fluoresceine indocyanine green angiography^10^. The reason for this is suspected to be a congestion of the vortex veins^11^, leading to their remodeling and causing increased hydrostatic pressure in the choroid. This in turn may result in the development of a pachychoroid. The congestion of the vortex veins is hypothesized to be a resultant of impeded vascular outflow due to thick sclera or increased scleral rigidity^12,13^. Recent studies showed an increased scleral thickness in CSCR patients compared to normal controls. Scleral fenestration consequently showed an improvement of the disease in one case of a chronic CSCR patient^14^.

Transferred to an increased incidence of pachychoroidal features in patients with RVO, one could possibly assume that the narrowed and impeded passage through a thickened and rigid pachysclera predisposes to the congestion of all ophthalmic veins, be it the vortex veins, leading to a pachychoroid, or the central retinal vein, leading to CRVO; and that these clinical pictures could therefore occur together.

Less likely, but also to be considered, choroidal flow changes secondary to changes in ocular arterial perfusion might partially explain a possible connection between pachychoroid disorders and RVO. Data from Abe et al. indicate that a reduction in choriocapillaris perfusion due to atherosclerosis or high sympathetic activity overproportionally increases choroidal blood flow^15^. Both atherosclerosis, which represents one of the major risk factors for RVO^1^, and high sympathetic activity, which has been shown to be involved in pachychoroid disorders^16^, induce reductions in choriocapillaris perfusion^15^. This consequently causes a secondary passive overflow into the surrounding choroidal veins along with a dilatation of the lumen and the formation of the so called pachyvessels^15,17^.

Limitations of our study include its retrospective nature and the fact that choroidal thickness was not measured in eyes with RVO, but in their respective partner eyes. Since RVO however is known to deregulate ocular, including choroidal blood flow^18^, measurements in the same eye would have been confounded. Moreover, our study concept is supported by the bilaterality of pachychoroid disorders. Nagia et al. could detect choroidal thickening in both NAION and their respective partner eyes^3^, and Ersoz could detect a pachychoroid in 91.8% of partner eyes in cases of CSCR, with PPE in 61%^19^. Another limitation is that some studies do not define pachychoroid disease by increased SFCT only, as it depends on age and axial length. For this reason, we matched both groups in terms of age and spherical equivalent. Also, the significantly increased number of symptomatic pachychoroid in the RVO group further supports the hypothesis that the thickened choroid in the RVO group might be pathological.

## Conclusions

In conclusion, our data indicate a higher SFCT and higher incidence of pachychoroid disorders in patients with RVO. Pachychoroid might therefore be a risk factor for RVO or share risk factors with RVO. Both entities might share similar pathophysiologic mechanisms.

Since a complementary therapy of pachychoroid could possibly also optimize the long-term management of RVO, further prospective studies are necessary to clearly clarify a possible correlation.
